# Safety, Pharmacokinetics, and Antiviral Activity of a Novel HIV Antiviral, ABX464, in Treatment-Naive HIV-Infected Subjects in a Phase 2 Randomized, Controlled Study

**DOI:** 10.1128/AAC.00545-17

**Published:** 2017-06-27

**Authors:** Jean-Marc Steens, Didier Scherrer, Paul Gineste, P. Noel Barrett, Supparatpino Khuanchai, Ratanasuwan Winai, Kiat Ruxrungtham, Jamal Tazi, Robert Murphy, Hartmut Ehrlich

**Affiliations:** a Abivax, Paris, France; b Independent Consultant, c/o Abivax, Paris, France; c Department of Medicine, Chiang Mai University, Chiang Mai, Thailand; d Department of Preventive and Social Medicine, Siriraj Hospital, Mahidol University, Bangkok, Thailand; e HIV Netherlands Australia Thailand Research Collaboration (HIV-NAT), Chulalongkorn University Hospital, Bangkok, Thailand; f Institut de Genetique Moleculaire, University of Montpellier, Montpellier, France; g Northwestern University Feinberg School of Medicine, Chicago, Illinois, USA

**Keywords:** ABX464, human immunodeficiency virus

## Abstract

We investigated the safety and antiviral effects of an anti-HIV compound (ABX464) with a unique mechanism of viral replication inhibition. This was a randomized, double-blind, placebo-controlled, dose-ranging study in treatment-naive HIV-infected patients. Participants were assigned to eight groups; each group included eight subjects receiving either the study compound, ABX464 (*n* = 6), or the corresponding placebo (*n* = 2), according to a randomization code. The first dose administered was 25 mg, given once or 3 times a day over a 2- to 3-week period. Ascending doses of up to 150 mg were delivered after review of the safety data. The primary objective of the study was to assess the safety and tolerability of ABX464 after repeated oral administrations in subjects infected by HIV. Sixty-six subjects were enrolled and were randomized. Sixty-three subjects completed the study according to the study protocol. Twenty-one adverse events (AEs) were reported by 7 subjects out of 16 (44%) who received placebo, and 158 AEs were reported by 39 subjects out of 50 (78%) who received the study drug. In the ABX464 treatment group, all of these adverse events were mild to moderate. No subjects discontinued treatment due to drug-related AEs. Administration of ABX464 at up to 150 mg once a day was safe and well tolerated in HIV-infected subjects. An efficacy signal with respect to a reduction of the viral load by ABX464 was detected, mainly in subjects treated at the highest dose. Further studies will be required to demonstrate antiviral effects in HIV-infected subjects in combination with other antiretroviral therapies. (This study is registered on the ClinicalTrials.gov website under registration no. NCT02452242.)

## INTRODUCTION

The introduction of combination antiretroviral therapy (cART) has led to a major reduction in HIV-related mortality and morbidity, with millions of AIDS-related deaths having been prevented in the recipients of this therapy (1). The combination of HIV protease, integrase, and reverse transcriptase inhibitors has dramatically changed the prognosis of HIV infection, so that as a result, HIV is now considered a chronic disease in many countries (2). Despite these substantial therapeutic advances, HIV infection cannot be cured and lifelong cART is required to keep HIV infection in check. This is due to the existence of HIV reservoirs in some long-lived cell populations that harbor integrated latent HIV provirus, as well as the persistence of HIV in anatomical reservoirs (3–5). The interruption of therapy results in the virus rapidly rebounding to pretreatment levels (6).

Even with the major successes of cART, there are still substantial deficiencies in the available therapies, so that as the HIV epidemic approaches its fourth decade, effective therapy and prevention remain elusive, especially in the countries most affected by the virus (7). The achievement of either a functional cure (long-term control of HIV in the absence of cART) or a sterilizing cure (elimination of all HIV-infected cells) still remains an important therapeutic objective (8).

A unique feature of the HIV replication cycle has been utilized to generate a compound with a unique mechanism of viral replication inhibition (9). This inhibition is achieved by promoting splicing of viral RNA and thereby interfering with the production of the essential regulatory proteins Rev and Tat or by inhibiting the export of viral mRNA required for Gag, Pol, and Env production and for generation of genomic RNA. ABX464 targets the interaction of Rev with cellular ribonucleoprotein complexes, an interaction which is a step essential for virus replication and, due to this specificity, does not impact normal cellular splicing (9–12).

ABX464 has been demonstrated to be effective in inhibiting the replication of different HIV subtypes in peripheral blood mononuclear cells (PBMCs) and macrophages and did not induce any drug resistance during up to 24 weeks of treatment *in vitro*. ABX464 also substantially reduced virus replication in two humanized mouse models of HIV infection. More importantly, while the viral load already increased dramatically less than 2 weeks after the termination of cART treatment in control animals, a substantially lower virus rebound was observed after the termination of therapy in the ABX464-treated animal group. This antiviral effect was sustained over the full 6 weeks of virus load measurements after treatment termination (9).

A first-in-human study has been completed in healthy subjects to determine the pharmacokinetic (PK) and safety profiles of a single ascending oral dose of ABX464. This study confirmed that ABX464 is well tolerated and rapidly and substantially metabolized into ABX464-*N*-glucuronide (ABX464-NGlc) in human subjects (13).

A second study in healthy volunteers to evaluate the impact of food consumption and repeated dosing on ABX464 pharmacokinetic properties and biological safety has also been completed, and this has confirmed the good tolerability profile of the product (14). The present study (ClinicalTrials.gov registration no. NCT02452242) was initiated with the primary objective to assess the safety and the tolerability of ABX464 after repeated oral administrations in patients infected by HIV and with the secondary objective to determine the effect on the HIV viral load of a repeated dose of ABX464.

## RESULTS

### Baseline characteristics and patient disposition.

**FIG 1:**
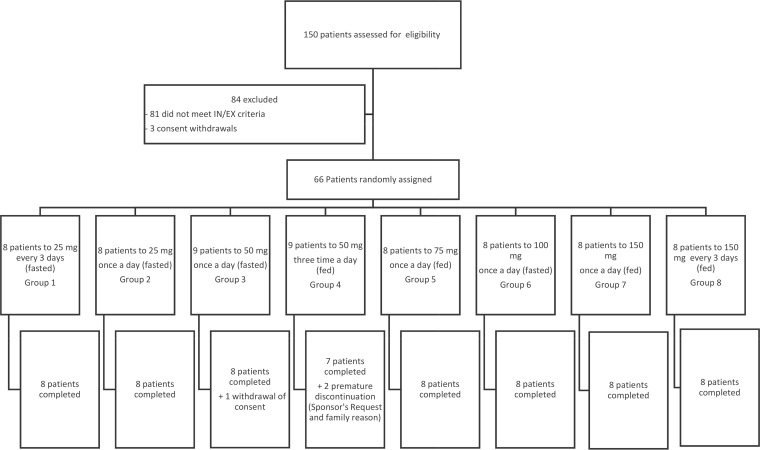
Study profile. IN/EX, inclusion/exclusion.

A total of 150 subjects were assessed for eligibility. A total of 66 subjects who were infected by HIV-1 or HIV-2, naive or within the therapeutic window, and male or female aged between 18 and 66 years and who had a body mass index (BMI) in the range from 17.0 to 33.3 kg/m^2^ were enrolled and were randomized into this study (Fig. 1). Over the treatment phase, three subjects were prematurely withdrawn from the study for non-drug-related reasons. One subject withdrew his consent due to the difficulty in performing blood sampling (his veins were hardly accessible). One subject discontinued the study per the sponsor's request; the subject was concomitantly treated with methadone, which was not prohibited at the beginning of the study but which was prohibited following discussions with the French Competent Authority (methadone is a CYP1A2 inducer). These two patients were replaced. The third subject exited the study after 3 weeks of treatment for family reasons and was not replaced.

Sixty-three subjects completed the study according to the study protocol and their dose regimen. Their demographic characteristics (age, weight, and BMI) are presented in Table 1. Overall, 40 (60.6%) of the study subjects were male. Males and females were enrolled in each of the dose groups except in the group receiving 150 mg every 3 days, in which all subjects were male. The subjects were mainly Caucasian at the Mauritius site, while all subjects enrolled in Thailand were Asian.

**TABLE 1:** Age, weight, and BMI at the baseline (day −1) by group

Group	Value	Age (yr)			Wt (kg)			BMI (kg/m^2^)		
		Placebo	ABX464	Total	Placebo	ABX464	Total	Placebo	ABX464	Total
Group 1	No. of subjects	2	6	8	2	6	8	2	6	8
	Mean ± SD	32.0 ± 18.4	41.2 ± 17.3	38.9 ± 16.7	64.60 ± 15.27	64.97 ± 9.14	64.88 ± 9.64	25.80 ± 2.69	23.30 ± 3.65	23.93 ± 3.45
	Min., max.^*a*^	19, 45	18, 66	18, 66	53.8, 75.4	48.0, 71.0	48.0, 75.4	23.9, 27.7	17.6, 26.4	17.6, 27.7
Group 2	No. of subjects	2	6	8	2	6	8	2	6	8
	Mean ± SD	23.0 ± 2.8	39.8 ± 14.8	35.6 ± 14.8	62.50 ± 10.61	76.73 ± 18.88	73.18 ± 17.72	20.35 ± 4.74	28.07 ± 4.63	26.14 ± 5.59
	Min., max.	21, 25	24, 65	21, 65	55.0, 70.0	47.0, 103.0	47.0, 103.0	17.0, 23.7	19.6, 33.3	17.0, 33.3
Group 3	No. of subjects	2	7	9	2	7	9	2	7	9
	Mean ± SD	33.0 ± 17.0	29.3 ± 8.2	30.1 ± 9.4	65.85 ± 17.89	62.40 ± 7.93	63.17 ± 9.46	23.60 ± 7.35	23.59 ± 4.09	23.59 ± 4.39
	Min., max.	21, 45	19, 37	19, 45	53.2, 78.5	50.0, 74.0	50.0, 78.5	18.4, 28.8	17.5, 28.0	17.5, 28.8
Group 4	No. of subjects	2	7	9	2	7	9	2	7	9
	Mean ± SD	30.5 ± 0.7	33.1 ± 7.3	32.6 ± 6.4	66.00 ± 4.24	61.57 ± 9.55	62.56 ± 8.63	25.35 ± 2.33	22.53 ± 3.56	23.16 ± 3.43
	Min., max.	30, 31	24, 46	24, 46	63.0, 69.0	44.0, 72.0	44.0, 72.0	23.7, 27.0	17.9, 28.1	17.9, 28.1
Group 5	No. of subjects	2	6	8	2	6	8	2	6	8
	Mean ± SD	33.5 ± 6.4	30.0 ± 9.5	30.9 ± 8.6	51.20 ± 10.75	56.07 ± 8.38	54.85 ± 8.47	19.35 ± 2.05	20.80 ± 3.51	20.44 ± 3.14
	Min., max.	29, 38	20, 46	20, 46	43.6, 58.8	47.6, 72.0	43.6, 72.0	17.9, 20.8	17.5, 25.3	17.5, 25.3
Group 6	No. of subjects	2	6	8	2	6	8	2	6	8
	Mean ± SD	38.0 ± 21.2	43.3 ± 8.0	42.0 ± 10.8	54.40 ± 9.33	59.17 ± 17.60	57.98 ± 15.45	20.70 ± 4.38	21.47 ± 4.04	21.28 ± 3.81
	Min., max.	23, 53	30, 54	23, 54	47.8, 61.0	41.0, 89.0	41.0, 89.0	17.6, 23.8	17.7, 28.7	17.6, 28.7
Group 7	No. of subjects	2	6	8	2	5	7^*b*^	2	5	7^*b*^
	Mean ± SD	22.0 ± 0.0	29.3 ± 10.0	27.5 ± 9.1	65.90 ± 16.40	68.62 ± 16.16	67.84 ± 14.86	22.25 ± 6.43	23.28 ± 3.48	22.99 ± 3.90
	Min., max.	22, 22	19, 46	19, 46	54.3, 77.5	49.5, 92.6	49.5, 92.6	17.7, 26.8	18.0, 27.6	17.7, 27.6
Group 8	No. of subjects	2	6	8	2	6	8	2	6	8
	Mean ± SD	34.5 ± 4.9	23.5 ± 4.4	26.3 ± 6.6	68.05 ± 4.31	61.07 ± 6.46	62.81 ± 6.55	23.40 ± 1.70	20.45 ± 2.90	21.19 ± 2.88
	Min., max.	31, 38	19, 31	19, 38	65.0, 71.1	54.2, 70.0	54.2, 71.1	22.2, 24.6	17.9, 26.0	17.9, 26.0

a Min., minimum value; max., maximum value.

b The value was missing for one subject.

All baseline characteristics were collected during the screening visit and the day 0 visit. None of the enrolled subjects exhibited clinical or biological abnormalities which prevented them from being included. All out-of-range values observed for electrocardiogram (ECG) parameters or vital signs were not clinically significant. Except for the CD4 and CD8 T-cell counts (consequences of the active HIV infection), all abnormal biochemistry or hematology values were not clinically significant except in 2 subjects. One had an elevated gamma-glutamyl transferase level (80 U/liter) at screening, but this was assessed to be not clinically significant by the investigator. Moreover, the subject had a reduced hemoglobin level (10 g/dl) at screening, but on day 1, the level increased to 11.6 g/dl (not clinically significant). The second subject had elevated aspartate aminotransferase and alanine aminotransferase levels at screening (235 and 157 U/liter, respectively), but these values were back to normal on day 0 (predose time point). Moreover, the subject also had a low platelet count at screening but normal values on day 0.

### Safety and tolerability.

The frequency and severity of the most frequent treatment-emergent adverse events (TEAEs) are presented in Table 2. A total of 179 adverse events (AEs) were reported in 46 subjects out of 66 (70%); 21 AEs were reported by 7 subjects out of 16 (44%) who received placebo, and 158 AEs were reported by 39 subjects out of 50 (78%) who received the study drug, regardless of the dose or the drug regimen. In the ABX464 treatment group, all of these adverse events were mild to moderate, and none of the TEAEs led to treatment discontinuation. There was only 1 serious adverse event (SAE) reported by a subject, who had a left leg fracture. This event was the result of a motorbike accident. This subject received placebo.

**TABLE 2:** Subjects experiencing at least one TEAE of mild or moderate intensity by SOC, PT, and group regardless of causal relationship

AE	No. (%) of subjects experiencing at least one TEAE																	
	Placebo (*n* = 16)		Group 1 (*n* = 6)		Group 2 (*n* = 6)		Group 3 (*n* = 7)		Group 4 (*n* = 7)		Group 5 (*n* = 6)		Group 6 (*n* = 6)		Group 7 (*n* = 6)		Group 8 (*n* = 6)	
	Mild	Moderate	Mild	Moderate	Mild	Moderate	Mild	Moderate	Mild	Moderate	Mild	Moderate	Mild	Moderate	Mild	Moderate	Mild	Moderate
Total	7 (43.8)	1 (6.3)	2 (33.3)	2 (33.3)	2 (33.3)	0 (0.0)	2 (28.6)	1 (14.3)	7 (100.0)	1 (14.3)	6 (100.0)	1 (16.7)	6 (100.0)	3 (50.0)	5 (83.3)	4 (66.7)	6 (100.0)	1 (16.7)
Gastrointestinal disorders	3 (18.8)	1 (6.3)	2 (33.3)	0 (0.0)	2 (33.3)	0 (0.0)	1 (14.3)	0 (0.0)	3 (42.9)	1 (14.3)	5 (83.3)	1 (16.7)	2 (33.3)	1 (16.7)	0 (0.0)	3 (50.0)	6 (100.0)	0 (0.0)
Nausea	1 (6.3)	0 (0.0)	1 (16.7)	0 (0.0)	0 (0.0)	0 (0.0)	1 (14.3)	0 (0.0)	0 (0.0)	0 (0.0)	3 (50.0)	1 (16.7)	0 (0.0)	0 (0.0)	4 (66.7)	3 (50.0)	6 (100.0)	0 (0.0)
Vomiting	0 (0.0)	0 (0.0)	1 (16.7)	0 (0.0)	2 (33.3)	0 (0.0)	1 (14.3)	0 (0.0)	1 (14.3)	0 (0.0)	4 (66.7)	0 (0.0)	0 (0.0)	0 (0.0)	1 (16.7)	3 (50.0)	4 (66.7)	0 (0.0)
Upper abdominal pain	0 (0.0)	0 (0.0)	0 (0.0)	0 (0.0)	0 (0.0)	0 (0.0)	0 (0.0)	0 (0.0)	2 (28.6)	1 (14.3)	0 (0.0)	0 (0.0)	2 (33.3)	1 (16.7)	0 (0.0)	0 (0.0)	0 (0.0)	0 (0.0)
Diarrhea	2 (12.5)	1 (6.3)	0 (0.0)	0 (0.0)	1 (16.7)	0 (0.0)	0 (0.0)	0 (0.0)	0 (0.0)	0 (0.0)	1 (16.7)	0 (0.0)	0 (0.0)	0 (0.0)	0 (0.0)	0 (0.0)	0 (0.0)	0 (0.0)
Abdominal pain	0 (0.0)	0 (0.0)	0 (0.0)	0 (0.0)	0 (0.0)	0 (0.0)	0 (0.0)	0 (0.0)	0 (0.0)	0 (0.0)	0 (0.0)	0 (0.0)	0 (0.0)	0 (0.0)	0 (0.0)	0 (0.0)	1 (16.7)	0 (0.0)
Nervous system disorders	4 (25.0)	0 (0.0)	0 (0.0)	0 (0.0)	1 (16.7)	0 (0.0)	1 (14.3)	1 (14.3)	2 (28.6)	0 (0.0)	5 (83.3)	0 (0.0)	5 (83.3)	0 (0.0)	4 (66.7)	4 (66.7)	6 (100.0)	0 (0.0)
Headache	4 (25.0)	0 (0.0)	0 (0.0)	0 (0.0)	1 (16.7)	0 (0.0)	1 (14.3)	1 (14.3)	2 (28.6)	0 (0.0)	5 (83.3)	0 (0.0)	5 (83.3)	0 (0.0)	4 (66.7)	3 (50.0)	6 (100.0)	0 (0.0)
Musculoskeletal and connective tissue disorders	1 (6.3)	0 (0.0)	0 (0.0)	1 (16.7)	1 (16.7)	0 (0.0)	1 (14.3)	0 (0.0)	3 (42.9)	0 (0.0)	0 (0.0)	0 (0.0)	0 (0.0)	0 (0.0)	1 (16.7)	0 (0.0)	1 (16.7)	0 (0.0)
Arthralgia	0 (0.0)	0 (0.0)	0 (0.0)	0 (0.0)	0 (0.0)	0 (0.0)	1 (14.3)	0 (0.0)	1 (14.3)	0 (0.0)	0 (0.0)	0 (0.0)	0 (0.0)	0 (0.0)	1 (16.7)	0 (0.0)	0 (0.0)	0 (0.0)
Back pain	0 (0.0)	0 (0.0)	0 (0.0)	0 (0.0)	1 (16.7)	0 (0.0)	0 (0.0)	0 (0.0)	2 (28.6)	0 (0.0)	0 (0.0)	0 (0.0)	0 (0.0)	0 (0.0)	0 (0.0)	0 (0.0)	0 (0.0)	0 (0.0)
Pain in extremity	0 (0.0)	0 (0.0)	0 (0.0)	0 (0.0)	0 (0.0)	0 (0.0)	0 (0.0)	0 (0.0)	1 (14.3)	0 (0.0)	0 (0.0)	0 (0.0)	0 (0.0)	0 (0.0)	0 (0.0)	0 (0.0)	1 (16.7)	0 (0.0)
Flank pain	1 (6.3)	0 (0.0)	0 (0.0)	0 (0.0)	0 (0.0)	0 (0.0)	0 (0.0)	0 (0.0)	0 (0.0)	0 (0.0)	0 (0.0)	0 (0.0)	0 (0.0)	0 (0.0)	0 (0.0)	0 (0.0)	0 (0.0)	0 (0.0)
Sciatica, back pain	0 (0.0)	0 (0.0)	0 (0.0)	1 (16.7)	0 (0.0)	0 (0.0)	0 (0.0)	0 (0.0)	0 (0.0)	0 (0.0)	0 (0.0)	0 (0.0)	0 (0.0)	0 (0.0)	0 (0.0)	0 (0.0)	0 (0.0)	0 (0.0)
Neck pain	0 (0.0)	0 (0.0)	0 (0.0)	0 (0.0)	0 (0.0)	0 (0.0)	0 (0.0)	0 (0.0)	1 (14.3)	0 (0.0)	0 (0.0)	0 (0.0)	0 (0.0)	0 (0.0)	0 (0.0)	0 (0.0)	0 (0.0)	0 (0.0)
Injury, poisoning, and procedural complications	0 (0.0)	0 (0.0)	0 (0.0)	0 (0.0)	0 (0.0)	0 (0.0)	0 (0.0)	0 (0.0)	0 (0.0)	0 (0.0)	0 (0.0)	0 (0.0)	0 (0.0)	0 (0.0)	0 (0.0)	0 (0.0)	0 (0.0)	1 (16.7)
Skin abrasion	0 (0.0)	0 (0.0)	0 (0.0)	0 (0.0)	0 (0.0)	0 (0.0)	0 (0.0)	0 (0.0)	0 (0.0)	0 (0.0)	0 (0.0)	0 (0.0)	0 (0.0)	0 (0.0)	0 (0.0)	0 (0.0)	0 (0.0)	1 (16.7)

The most frequently reported TEAEs were headache, nausea, vomiting, and upper abdominal pain. Forty-six episodes of headache were reported in 32 subjects (48.5%) overall.

Twenty-five percent of the subjects in the placebo group and 56% of the subjects in the ABX464 treatment groups reported at least one episode of headache. The frequency of headache in the groups receiving ABX464 at 25 mg and 50 mg compared favorably to that in the placebo group, even in the group receiving ABX464 at 50 mg three times a day (t.i.d.), while the headache frequency increased to between 83.3% and 100% in the higher-dose groups. This pattern indicated a dose relationship in the occurrence of headache starting from the dose of 75 mg once a day (q.d.) onwards. The episodes of headache were generally of mild intensity (40 episodes), with 6 reports of headache of moderate intensity. Headaches generally occurred on the first day of administration (about 40%) but also occurred later during the course of treatment. Episodes of headache were generally of a relatively short duration, and most of them resolved spontaneously or with symptomatic treatments.

Twenty-five cases of nausea were reported by 18 subjects (27.2%) overall. Six percent of the subjects in the placebo group and 42.5% of the subjects in the ABX464 treatment groups reported at least one episode of nausea. Nausea was rarely reported by subjects in the 25-mg or 50-mg dose groups under the fed condition. Its frequency increased according to the dose level (66.7% and 100%, respectively, in the groups receiving ABX464 at 75 mg and 150 mg q.d.). Nausea was experienced on the first day of administration in about half of the cases. Most of the cases of nausea were considered related to ABX464 (23 out of 25 cases). They were of mild intensity in most of the cases (20 cases), with sporadic episodes of nausea of moderate intensity being noted.

Twenty-five cases of vomiting were reported by 16 subjects (32%) overall. Four of the vomiting episodes were considered unlikely related to ABX464. Vomiting episodes were experienced throughout the treatment duration but in half of the cases were experienced on the first day of treatment only. They were of mild intensity in almost all cases (3 cases were of moderate intensity). No vomiting episodes were observed in subjects receiving placebo.

The number of vomiting episodes was higher in the group receiving 150 mg ABX464 (66.7% of the subjects), and the incidence of vomiting did not significantly decrease when ABX464 was administered at 150 mg every 3 days (50.0% of the subjects) but was clearly reduced when ABX464 was given according to a 50-mg t.i.d. regimen. Twenty cases of upper abdominal pain were reported by 4 subjects (6%) overall. The intensity of upper abdominal pain was equally split between mild and moderate. No cases of upper abdominal pain were observed in subjects receiving placebo.

A skin rash episode was reported in two subjects receiving ABX464 at 75 mg q.d. under fed conditions, with one episode being of moderate intensity. Both events occurred on the same day that the subjects were at the hospital unit and were associated with body ache. The events were probably related to the study drug according to the investigator. These events did not lead to treatment interruption and resolved without sequelae with symptomatic treatment.

### Pharmacokinetics.

This study consisted of eight groups containing a total of 66 subjects, with 50 of them receiving an active treatment. Considering that there were 3 different dosing regimens and 2 different conditions of administration, the opportunity to carry out formal statistical analysis was found to be limited to dose proportionality assessment on day 1 for ABX464. All the other evaluations were based on comparisons between individual data and descriptive statistics.

The data obtained for both ABX464 and ABX464-NGlc in HIV-infected subjects demonstrated no differences in key PK characteristics compared to those previously reported in healthy non-HIV-infected subjects (13, 14). ABX464 was rapidly eliminated with a half-life (*t*_1/2_) of about 1 h, while its metabolite, ABX464-NGlc, was very slowly cleared with a *t*_1/2_ of about 100 h. Data comparing the key PK characteristics of ABX464 for one dose regimen in HIV-infected subjects from this study and healthy non-HIV-infected subjects from a previous study (13) are presented in Table S1 in the supplemental material. As previously reported for non-HIV-infected subjects, a concomitant food effect led to a substantial increase in the relative bioavailability of ABX464, but the food-induced increase in the level of ABX464 exposure did not lead to an increase in the level of ABX464-NGlc exposure of a similar magnitude (data not shown). No substantial differences in key PK characteristics according to the ethnic origins of the subjects were observed.

### Viral load.

**FIG 2:**
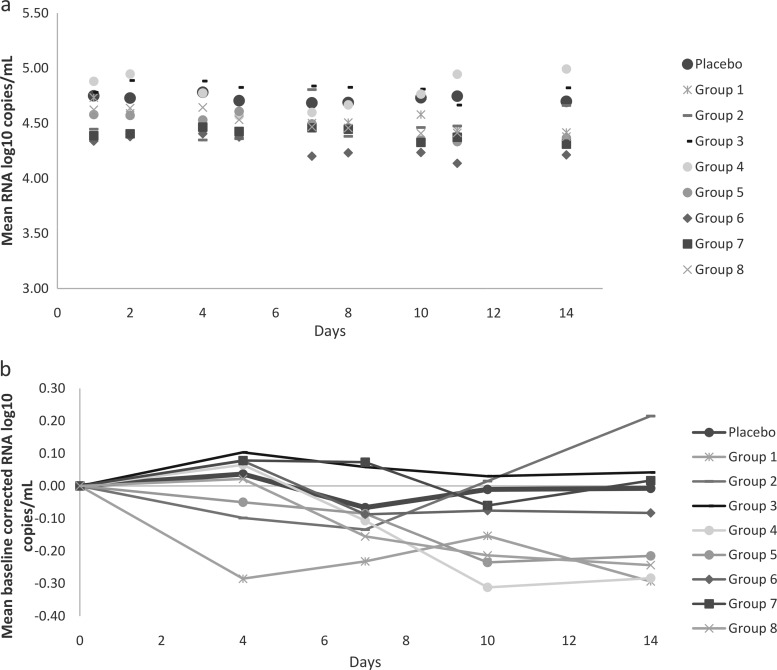
(a) Mean log_10_ number of RNA copies per milliliter versus time after multiple oral administrations of ABX464; (b) mean baseline-corrected log_10_ number of RNA copies per milliliter versus time after multiple oral administrations of ABX464.

The mean values of the log_10_-transformed number of HIV-1 RNA copies per milliliter for each group, including the placebo group, over a standardized study treatment duration of 14 days are presented in Fig. 2a. The data for all subjects who received placebo were pooled. The baseline-corrected data for all groups are presented in Fig. 2b. A longitudinal analysis for changes in the viral load compared to those at the baseline was also carried out. The active-treatment groups were compared to the placebo group, with placebo data from each of the eight groups being pooled. No statistically significant differences in the mean changes in the viral load between the different treatment groups and the placebo group were seen for any time point over the 14-day study period.

The effect of ABX464 on the viral load was further investigated in an experimental analysis by focusing on data from individual subjects, i.e., determining the number of responder subjects on the basis of a responder definition of a decrease in the viral load from that at the baseline of at least 0.5 log_10_ at at least one time point during the study. The changes for subjects with at least one treatment response over the entire study treatment are presented in Table 3. A total of 14/50 subjects (28%) in total (3 in group 1, 1 in group 2, 2 in group 4, 1 in group 5, 2 in group 6, 1 in group 7, and 4 in group 8) demonstrated a decrease of ≥0.5 log_10_ in their HIV RNA load over that period. A total of 49 measurements among the ABX464-treated subjects demonstrated a 0.5 log_10_ decrease over the treatment period, while 3 of 16 (18.7%) placebo-treated subjects demonstrated a ≥0.5 log_10_ reduction in the HIV RNA load over the treatment period, with just 3 measurements among the subjects in the placebo group showing this decrease in the viral load (Fisher's exact test, *P* = 0.0004). From day 29 to day 70, a total of 11 subjects demonstrated a decrease in the viral load of ≥0.5 log_10_. All of these, except for one subject who had a decrease of 0.79 log_10_ at day 56 only, had already demonstrated a treatment response in the first 28 days.

**TABLE 3:** Individual change in log_10_ number of RNA copies per milliliter from that at the baseline of <−0.5 log by visit over the ABX464 treatment period

Group	Subject	Day	Change in log_10_ no. of RNA copies/ml from that at the baseline
1	102	4	−0.99
		5	−1.47
		7	−1.36
		8	−1.57
		10	−1.43
		11	−1.61
		13	−1.4
		14	−1.51
	105	14	−0.5
		16	−0.52
		17	−0.65
	106	5	−0.72
		11	−0.56
		13	−0.62
		16	−0.74
		17	−0.51
		19	−0.61
2	201	14	−0.54
4	2007	10	−0.62
	2009	10	−0.72
		14	−1.23
		18	−1.39
5	605	2	−0.71
		4	−0.77
		5	−0.76
		7	−0.89
		8	−0.83
		10	−1.06
		11	−1.05
6	804	11	−0.52
		15	−0.59
	807	11	−0.51
		18	−1.44
7	908	16	−0.72
		19	−0.53
		20	−0.59
8	1001	10	−0.5
		11	−0.62
		14	−0.72
		15	−0.68
	1004	10	−0.5
		14	−0.51
		15	−0.51
	1005	11	−0.52
		7	−0.53
	1008	8	−0.59
		10	−0.57
		11	−0.56
		14	−0.53

**FIG 3:**
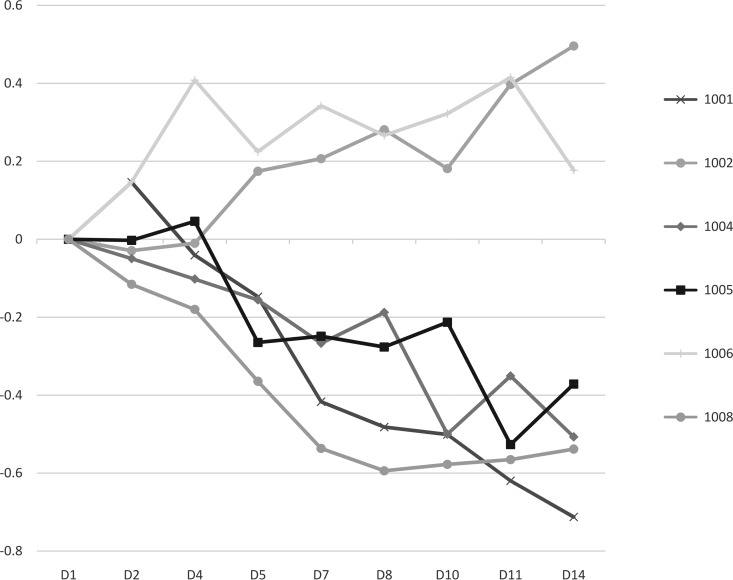
Individual change in the log_10_ number of RNA copies per milliliter from that at the baseline by visit over the 2 weeks of ABX464 treatment (group 8). D, day.

The highest number of responders (4 of 6) was seen in group 8 (Table 3), with the strongest indication of an antiviral response being seen in this group that received the highest ABX464 dosage (150 mg once a day for 2 weeks). Figure 3 shows the consistent reduction in the HIV RNA load from the baseline in 4 of 6 treated subjects over the 14-day treatment period. The trend toward an efficacy signal at the highest dose was also confirmed by the longitudinal analysis of the viral load data. The mixed model analysis of the RNA load from day 2 to day 14 for this group approached significance, with *P* being equal to 0.096 (Table S2). This compares to a *P* value of 0.2 calculated for the group receiving ABX464 at a dose of 50 mg t.i.d.

### T-cell analysis.

In addition to pharmacodynamic (PD) effects on the viral load, the study also included an analysis of the effect of the drug on CD4 and CD8 cell counts. For each ABX464 dose as well as for placebo, the CD4 profile showed no trend associated with product use or dose. Mean changes in the CD8 cell counts from those at the baseline also did not show any particular trend, but in most cases, mean values were stable over the period of measurement.

## DISCUSSION

One of the main challenges in the fight against HIV infection is the development of strategies that are able to eliminate the persistent viral reservoir that harbors integrated, replication-competent provirus within host cellular DNA (15). This reservoir is resistant to standard therapy, and viruses originating from this reservoir lead to rebound viremia once treatment is stopped (16, 17). Current antiviral treatment regimens contain at least two and preferably three drugs from two or more classes with the aim of suppressing viral replication. These regimens generally include reverse transcriptase, integrase, and protease inhibitors. The development of new therapeutic regimens is focused on improving safety, tolerability, and the resistance profile of drug combinations within the existing classes, with few ongoing efforts being made to develop novel drugs with the potential to eradicate the virus reservoir (18).

This study demonstrated that a first-in-class antiviral chemical entity, ABX464, with a novel mechanism of activity (9) is safe and has a good tolerability profile in HIV-infected subjects. An efficacy signal with respect to reduction of the viral load by ABX464 was detected, mainly in subjects treated with the highest dose.

The safety and tolerability profile of ABX464 in this HIV-infected subject population was similar to that previously reported for the product in two studies in healthy noninfected subjects. In this study, no product-related SAEs occurred, and no AEs led to premature discontinuation. The most common ABX464-related TEAEs were headaches, nausea, and vomiting. All of these reactions were either mild or moderate. Twenty cases of upper abdominal pain were reported by 4 subjects who received ABX464, but it should be noted that these were reported only from the Mauritius site and these cases should be considered with caution due to possible confusion between the verbatim nausea and upper abdominal pain. The frequency of these adverse reactions seemed to be dose related from the 75-mg dose upwards (Table 2). The frequency of subjects receiving ABX464 who reported TEAEs varied between 33% and 50% in groups 1 to 3, while it reached 100% in all other groups. The adverse reactions also seemed to be closely related to the ABX464 peak exposure, as a food effect has been observed and because the 50-mg t.i.d. regimen (group 4) tended to decrease the occurrence of these adverse events.

Although all reactions were either mild or moderate, the frequency of AEs was relatively high. Currently licensed antiretroviral compounds are very well tolerated, and further extensive studies with ABX464 will be required to confirm the tolerability of this compound, especially when used in combination with other antiretroviral therapies.

With respect to other safety parameters, no clinically significant abnormalities in electrocardiogram (ECG) parameters were reported, and none of the changes in any laboratory parameter observed between baseline and posttreatment measurements suggested an impact of ABX464 treatment. The great majority of discrepancies measured were not clinically significant.

The pharmacokinetic profiles of ABX464 and its main metabolite, ABX464-NGlc, have previously been extensively studied but only in healthy, non-HIV-infected subjects (13, 14). This study demonstrated that there were no substantial differences in the PK characteristics of the two compounds between HIV-infected and non-HIV-infected subjects. It was also confirmed that the food effect of both compounds was comparable to that reported in noninfected subjects (14).

Although the primary endpoint of this study was the safety profile of ABX464 in this subject population, i.e., HIV-infected persons, an analysis was also carried out to determine the effects of ABX464 on the viral load. An efficacy signal was detected especially at the highest dose (150 mg once a day), with 66% of the subjects treated with this dose but only 18.7% of the subjects in the pooled placebo group experiencing a decrease of at least 0.5 log_10_ for at least one measurement of the viral load over the 2-week treatment period. The evidence for this efficacy signal was also emphasized by the consistent reduction in the viral load compared to that at the baseline seen for four of the six subjects treated with this ABX464 regimen (Fig. 3) and by a *P* value approaching significance (despite the low subject numbers), which was obtained for the longitudinal analysis of the viral load for this group. Analysis of the demographic data and viral tropism (CCR5/CXCR4) for the six subjects provided no possible explanation why two subjects did not demonstrate a reduction in viral load at this dose. A possible dose effect could also be observed, especially for doses of 75 mg, 100 mg, and 150 mg once a day, with 16%, 33%, and 66% of the subjects receiving ABX464 at these three doses, respectively, demonstrating an antiviral response. The extent of this response can be compared to that seen for other novel anti-HIV drugs in early clinical trials when used as a monotherapy for short-term treatment. It has been reported that a novel antiviral that blocks HIV Vpu ion channel activity (19) had no significant effect on the HIV viral load in plasma or the CD4 T cell count. However, 10 days of treatment resulted in a 2.7-fold decrease or a 63% reduction in the mean total HIV-1 DNA copy number in the monocytes of treated individuals. Analysis of individual responses demonstrated that that three of the six treated individuals demonstrated significant reductions in total HIV-1 DNA levels within their monocytes (20).

The limited antiviral effect of ABX464 seen in this study is, however, not a surprising finding. This study involved a short-term treatment (14 or 28 days) with ABX464 as monotherapy. It has been reported that in humanized mouse studies, ABX464 induced a of a viral load reduction in infected mice with kinetics slower than those in mice treated with highly active antiretroviral therapy (HAART) (9). In these studies, whereas HAART was very efficient in reducing the viral load to undetectable levels after 2 to 3 weeks in all treated mice, only 30% of mice treated with ABX464 had an undetectable viral load after the same treatment period, and longer treatment with ABX464 was required to achieve a viral load reduction in more mice. In addition, the PK data reported here and in previous studies demonstrated that the parent compound has a very short half-life of approximately 1 h compared to the metabolite ABX464-NGlc, which has a *t*_1/2_ of approximately 100 h (13, 14). The maximum concentration in plasma (*C*_max_) of the metabolite was also approximately 160-fold higher than that of the parent compound. It has been demonstrated in *in vitro* studies that while ABX464 can inhibit HIV replication in PBMC and primary macrophage cultures, the metabolite demonstrated no antiviral effect on HIV in PBMCs. However, ABX464-NGlc was able to inhibit HIV replication in macrophage culture with the same 50% inhibitory concentration as the parent drug (9). Thus, it is unlikely that substantial effects on viral load, which are dependent on inhibition of replication of HIV on peripheral blood T cells, could be expected by use of ABX464 (which has such a short *t*_1/2_) as a monotherapy. It was concluded that treatment with ABX464 can result in the control of most of the cells contributing to viral rebound and that this control is due to the antiviral effects of the ABX464-NGlc metabolite on HIV replication in macrophages.

However, it is not possible to directly correlate antiviral effects with the bioavailability of the ABX464-NGlc metabolite. There are major differences in the metabolism of ABX464 between mice and humans, with ABX464-NGlc being only a minor metabolite in mice. The bioavailability of ABX464-NGlc was approximately 15-fold higher in the human subjects than in the mice, although the antiviral effects were substantially higher in the humanized mouse studies. Other mechanisms, possibly related to the immune-modulating effects of ABX464 (21), may be involved in the antiviral effects of ABX464 treatment. Additional studies to attempt to further elucidate the mechanism of action of ABX464 in humans are required and are currently ongoing.

Also, no studies were carried out in this trial to determine whether treatment resulted in the development of resistance to ABX464. However, extensive *in vitro* studies in human PBMCs demonstrated no development of resistance-inducing mutations after treatment with ABX464 for at least 24 weeks (9).

Thus, it is proposed that the optimal protocol for the use of ABX464 involves a combination therapy in which a standard HIV antiviral treatment is utilized to limit viral replication in the peripheral T cells and ABX464 is utilized to control viral replication and/or eliminate the virus from the reservoir macrophage population. Two phase 2a studies are currently ongoing to further investigate the safety, pharmacokinetics, and pharmacodynamics of ABX464 in combination with standard therapies. These include the use of ABX464 in combination with boosted darunavir or the use of ABX464 in subjects treated with dolutegravir or raltegravir combined with either tenofovir plus emtricitabine (TDF-FTC) or abacavir plus lamivudine (ABC-3TC). In conclusion, this study has demonstrated that ABX464 at doses of up to 150 mg once a day is safe and well tolerated in HIV-infected subjects. The antiviral activity seen in preclinical studies when ABX464 was used as a monotherapy could also be confirmed. These results support the continuing clinical development of ABX464 in HIV-infected subjects in combination with other antiretroviral therapies.

## MATERIALS AND METHODS

### Study design and participants.

This study was conducted between 27 January 2015 and 26 May 2016 at three centers in Thailand and one center in Mauritius. The clinical study (protocol no. ABX464-003), informed consent documents, and any other appropriate study-related documents were reviewed and approved by both the Clinical Research Regulatory Council, Mauritius, and the independent ethics committee (IEC) of CREC, Thailand. This was a randomized, multicenter, double-blind, placebo-controlled, ascending-dose-ranging phase 2a study in treatment-naive HIV-infected patients. The study population consisted of male or female patients who were infected by HIV-1 or HIV-2, treatment naive or within the therapeutic window, and 18 to 65 years of age and who had a body mass index (BMI) of 17 to 29 kg/m^2^. The other inclusion and exclusion criteria are described in Table S3 in the supplemental material.

### Randomization and masking.

Participants were recruited sequentially and randomly assigned to one of eight study groups. Each subject received an incremental identification number corresponding to the subject's rank of enrollment in the study. The treatment number was assigned to the subject according to the randomization list centrally generated by biostatistics. Participants and investigators were masked to the group allocation.

### Procedures.

Participants were assigned to one of eight groups; each group (with one exception) included eight subjects receiving either ABX464 (*n* = 6) or the corresponding placebo (*n* = 2) according to a randomization code. Dose selection was based on the first-in-human exploratory study conducted with ABX464, which showed that ABX464 is very well tolerated up to a dose of 150 mg once a day in non-HIV-infected subjects (13). In the present study with HIV-infected subjects, the first dose administered was 25 mg, given once or 3 times a day over a 2- to 3-week period. This initial dose was therefore considered safe for assessment of the safety and tolerability of ABX464 after repeated oral administrations in subjects infected by HIV and determination of the potential effect on the HIV viral load.

The final dosing protocol for each of the eight groups was as follows: group 1, 25 mg every 3 days for 2 to 3 weeks under fasted conditions; group 2, 25 mg once a day (q.d.) for 2 to 3 weeks under fasted conditions; group 3, 50 mg once a day for 2 to 3 weeks under fasted conditions; group 4, 50 mg 3 times a day (t.i.d.) for 4 weeks under fed conditions; group 5, 75 mg once a day for 2 weeks under fed conditions; group 6, 100 mg once a day for 2 to 3 weeks under fasted conditions; group 7, 150 mg every 3 days for 2 to 3 weeks under fed conditions; and group 8, 150 mg once a day for 2 weeks under fed conditions. Per the study protocol, the treatment period was defined as 2 weeks. However, if the study treatment was well tolerated (no safety issue), one additional week of treatment could be performed. The decision to proceed to the next highest dose was determined by the data and safety monitoring board after review of the laboratory safety data, vital signs, ECGs, and adverse event reports from the group receiving the current dose after 2 weeks of treatment.

A screening visit was carried out within 3 weeks prior to inclusion, and subjects who met all the inclusion criteria and none of the exclusion criteria were eligible for the inclusion visit on the day prior to drug administration. Final inclusion was done just before the first dosing on day 1.

Subjects were hospitalized from the day prior to the first dose to at least the morning of day 2. They were orally dosed with 25-mg capsules of ABX464 with 240 ml water under fed or fasted conditions. According to the group, subjects were dosed with 1, 2, 3, 4, or 6 capsules to deliver 25 mg, 50 mg, 75 mg, 100 mg, or 150 mg product, respectively, or the same number of placebo capsules.

Safety assessments and laboratory tests were carried out according to the schedules described in Table S4 for groups dosed every day, every 3 days, and 3 times a day. Follow-up visits were scheduled for 7 days after the last dose and subsequently once every week in order to assess the elimination of the main ABX464 metabolite and to assess the viral load. Follow-up visits were terminated when the virus load was within 0.5 log of the baseline value or after 3 follow-up visits, whichever came first. Prior to the statistical analyses, AEs were coded using the Medical Dictionary for Regulatory Activities (MedDRA dictionary; last available version) in order to be tabulated by system organ class (SOC) and preferred term (PT). The AEs were analyzed according to the treatment emergence phase from the first study drug administration on day 1 up to 48 h after the last study drug administration. The number and percentage of subjects with at least one TEAE were tabulated by SOC and PT for each group and dose. The number of occurrences of TEAEs was also analyzed in the same way by SOC and PT for each group and dose, by grade of severity for each group and dose, and by level of causality to the study drug for each group and dose.

Plasma samples were analyzed for ABX464 and ABX464-NGlc using validated bioanalytical methods by Atlanbio (Saint Nazaire, France) according to good laboratory practices. Pharmacokinetic parameters were calculated by noncompartmental analysis (NCA), using Phoenix WinNonlin software (Pharsight Corporation) running on a personal computer. On day 1, the following PK parameters were generated: *C*_max_, the time to *C*_max_ (*T*_max_), lag time (*t*_lag_), elimination rate constant (*k*_el_), area under the concentration-time curve (AUC) from time zero to time *t* (AUC_0–*t*_), AUC from time zero to infinity (AUC_0–∞_), apparent clearance (CL/*F*), and apparent volume of distribution (*V*/*F*). On the other days of PK assessment, only *C*_max_, *T*_max_, *t*_lag_, *k*_el_, and AUC_0–*t*_ were generated. The secondary analysis concerned pharmacodynamic (PD) activity, as expressed by CD4 and CD8 levels and viral load across time. The HIV viral load was analyzed in the prequalified individual local study center laboratories. CD4 and CD8 cell counts were also determined in the local study center laboratories. With respect to the viral load, changes in the log-transformed viral load values between 24 h postdose and predose were calculated on each day, and a comparison between each dose level and pooled placebo data was made. Moreover, the rate of success (defined as a decrease in the viral load value of at least 0.5 log) was evaluated for each treatment (including placebo) and compared between treatments.

Evaluation of a possible relationship between the viral load (or changes in the viral load) on a given day and the PK parameters of ABX464 and its main metabolites was visually investigated. Further evaluation of the relationship was then performed as an exploratory analysis according to the preliminary conclusions of the visual investigation.

### Statistical analysis.

No formal sample size determination was done. The aim of the study was to obtain reliable and consistent data and to expose a limited number of subjects to ABX464. To achieve this aim, six subjects per group received ABX464. A mixed model analysis of covariance of the changes in the viral load compared to that at the baseline was conducted on log-transformed values. Baseline parameters and age were covariates, treatment groups were fixed effects, and subjects were random effects.

## Supplementary Material

Supplemental material
