# Supplementary material for: Safety, Pharmacokinetics, and Antiviral Activity of a Novel HIV Antiviral, ABX464, in Treatment-Naive HIV-Infected Subjects in a Phase 2 Randomized, Controlled Study
Source: Antimicrob Agents Chemother. 2017 Jun 27;61(7):e00545-17. doi: 10.1128/AAC.00545-17 (PMC5487684; doi:10.1128/AAC.00545-17)
Supplement: Supplemental material [file supp_61_7_e00545-17__index.html]

Supplemental material 

# Safety, Pharmacokinetics, and Antiviral Activity of a Novel HIV Antiviral, ABX464, in Treatment-Naive HIV-Infected Subjects in a Phase 2 Randomized, Controlled Study

## Supplemental material

- Supplemental file 1 -

  Tables S1 to S4

  PDF, 403K
