# Supplementary material for: Safety, Pharmacokinetics, and Antiviral Activity of a Novel HIV Antiviral, ABX464, in Treatment-Naive HIV-Infected Subjects in a Phase 2 Randomized, Controlled Study
Source: Antimicrob Agents Chemother. 2017 Jun 27;61(7):e00545-17. doi: 10.1128/AAC.00545-17 (PMC5487684; doi:10.1128/AAC.00545-17)
Supplement: Supplemental material [file AAC.00545-17_zac007176341s1.pdf]

Table S1. Summary of descriptive statistics of ABX464 PK parameters after single oral administration of 50mg (fasted) ABX464 in Non-infected and HIV-infected subjects

**ABX464**		**C_{max} (ng/mL)**	**t_{max}[(1)] (h)**	**$AUC_{0-\infty}$ (ng.h/mL)**	**$t_{1/2}$ (h)**
Non-infected	**N**	23	23	17	17
	**Mean**	17.70	1.5	51.74	0.88
	**SD**	13.53	1.0–6.0	29.66	0.28
	**GM**	13.70	-	43.93	0.83
HIV-infected	**N**	7	7	7	7
	**Mean**	19.42	1.9	55.35	0.96
	**SD**	85.6	33.7	91.2	27.6
	**GM**	14.89	1.8	42.83	0.94

Table S2. Mixed Model Analysis: RNA Viral Load from Day 2 to Day 14
ABX464 (150 mg once a day) vs. Placebo
(ABX464)

Parameter	Category	Estimate	Standard Error	P-value
Intercept	NA	0.9499	0.3323	0.011
Treatment Group	Group 10 - 150 mg once a day	-0.1559	0.0884	0.096
Age	NA	-0.0042	0.0042	0.331
Visit	Day 2	0.0780	0.0558	0.172
	Day 4	0.1238	0.0550	0.031
	Day 5	0.0281	0.0547	0.609
	Day 7	-0.0076	0.0533	0.887
	Day 8	-0.0207	0.0520	0.692
	Day 10	0.0105	0.0488	0.831
	Day 11	0.0236	0.0444	0.597
	Day 13	0.0388	0.0646	0.550
Baseline	NA	-0.1855	0.0681	0.014

Table S3. Inclusion / Exclusion Criteria

Inclusion Criteria	Exclusion Criteria
Males or females, 18 to 65 years of age; infected by HIV-1 or HIV-2; with BMI between 17 and 29 kg/m^2; with CD4 cell count ≥ 350 /mm^3 and HIV RNA level between 5000-500,000 copies/mL; with clinical laboratory tests (hematology, blood chemistry, and urinalyses) within normal limits, or clinically acceptable<br>No current or prior antiretroviral treatment except for a brief time (*i.e.* less than 3 months). Potential antiretroviral agent should have been stopped at least 6 months prior to the screening visit.<br>Subjects with chronic hepatitis C were eligible for the study providing their liver function parameters are within the following ranges: platelets > 150,000/mm^3; alanine aminotransferase (ALAT), aspartate aminotransferase (ASAT) ≤ 3 ULN and gamma glutamyl transpeptidase (GGT) ≤ 2.5 × ULN; albumin > 40 g/L and providing that they were not receiving specific treatment during the study that could interfere with the study objectives.<br>Females were non-lactating and of non-childbearing potential or if of child bearing potential, had to have a effective double barrier contraceptive methods from at least two weeks prior to Day 1 until 3 months after the last dose of study medication;<br>Males had to comply with an effective barrier method of contraception from Day 1 until 3 months' days after the last dose of study medication.	Subjects with a history of any significant medical disorders which requires a physician's care; with history of any clinically significant local or systemic active infectious disease (other than HIV-1 or HIV-2 infection) within four weeks prior to drug administration; with any clinically significant laboratory abnormalities as defined as grade 2 or 3 in Common Terminology Criteria for AEs (CTC-AE); who have participated in a clinical trial of an investigational drug within 90 days prior to the start of the study.

Table S4. Schedule of Assessments

Days	Screening -21 à -2	D0	D7	D14	D21	D28	Weekly FU Visits
Informed Consent	X						
Physical Exam	X	X	X	X	X	X	
Study Drug dosing							
Serology (HBV, HCV, HIV)	X						
Hematology + Biochemistry (incl. Viral Load)	X	X	X	X	X	X	X
CD4 and CD8 count determination	X	X	X	X	X	X	
ECG (12 lead)	X	X				X	
Blood sample drug PK		X	X		X	X	X
